# Sweet Cherry Byproducts Processed by Green Extraction Techniques as a Source of Bioactive Compounds with Antiaging Properties

**DOI:** 10.3390/antiox9050418

**Published:** 2020-05-13

**Authors:** Luz Agulló-Chazarra, Isabel Borrás-Linares, Jesús Lozano-Sánchez, Antonio Segura-Carretero, Vicente Micol, María Herranz-López, Enrique Barrajón-Catalán

**Affiliations:** 1 Instituto de Biología Molecular y Celular (IBMC) and Instituto de Investigación, Desarrollo e Innovación en Biotecnología Sanitaria de Elche (IDiBE), Universitas Miguel Hernández (UMH), 03202 Elche, Spain; lagullo@umh.es (L.A.-C.); vmicol@umh.es (V.M.); e.barrajon@umh.es (E.B.-C.); 2 Research and Development Functional Food Centre (CIDAF), Health Science Technological Park, Avenida del Conocimiento 37, BioRegión Building, 18016 Granada, Spain; iborras@cidaf.es (I.B.-L.); jesusls@ugr.es (J.L.-S.); ansegura@ugr.es (A.S.-C.); 3 Department of Food Science and Nutrition, University of Granada, Campus of Cartuja, 18071 Granada, Spain; 4 Department of Analytical Chemistry, Faculty of Sciences, University of Granada, Avenida Fuentenueva s/n, 18071 Granada, Spain; 5 CIBER: CB12/03/30038 Fisiopatología de la Obesidad y la Nutrición, CIBERobn, Instituto de Salud Carlos III, 07122 Palma de Mallorca, Spain

**Keywords:** sweet cherry, byproduct, natural extract, antioxidant, cosmetic, HPLC-ESI-QTOF-MS

## Abstract

In the cosmetic industry, there is a continuous demand for new and innovative ingredients for product development. In the context of continual renovation, both cosmetic companies and customers are particularly interested in compounds derived from natural sources due to their multiple benefits. In this study, novel and green-extractive techniques (pressurized solvent, supercritical CO_2_, and subcritical water extractions) were used to obtain three new extracts from sweet cherry stems, a byproduct generated by the food industry. The extracts were characterized by high-performance liquid chromatography coupled to quadrupole-time-of-flight mass spectrometry (HPLC-ESI-QTOF-MS), and 57 compounds, mainly flavonoids but also organic and phenolic acids, fatty acids, and terpenes, were identified. After analytical characterization, a multistep screening approach, including antioxidant, enzymatic, and photoprotective cellular studies, was used to select the best extract according to its benefits of interest to the cosmetics industry. The extract obtained with supercritical CO_2_ presented the best characteristics, including a wide antioxidant capacity, especially against lipid peroxyl and ^•^OH free radicals, as well as relevant photoprotective action and antiaging properties, making it a potential new ingredient for consideration in the development of new cosmetics.

## 1. Introduction

Currently, society has a higher interest in skin care products obtained from natural sources compared to pharmaceutical compounds obtained by chemical synthesis [1]. Natural extracts have several advantages over synthetic compounds that make them desirable to the cosmetics industry, as society assumes that synthetic compounds can have harmful effects [2]. In addition, the food industry, including the sweet cherry (SC) industry, is characterized by the generation of a large amount of waste and byproducts that deserve better utilization for economic and environmental reasons. On the one hand, fruit wastes represent large economic losses and may present risks, such as greenhouse gas emissions in landfilling [3]; on the other hand, fruit byproducts are a valuable source of new ingredients for the food [4] and cosmetics [2] industries. The reduction of these food industry residues and byproducts and their revalorization as bioactive cosmetic ingredients contribute to reduce the ecological impact of these companies and is related to “UN 2030 Sustainable Development Goals” numbers 9 (industry, innovation, and infrastructures) and 12 (responsible consumption and production).

Natural extracts have been used traditionally in herbal medicines. The nutritional, chemopreventive, and pharmacological properties of natural extracts highlight the beneficial health effects of plant-derived compounds. In fact, extracts of many plant species have demonstrated antioxidant and anti-inflammatory capacities, and they can also present antitumoral, antimicrobial, or antiaging actions [5,6,7,8].

The biological activities of plant extracts are due to their high contents of potential bioactive compounds that can interact with different targets involved in molecular mechanisms related to alterations or diseases. These biological activities are mainly due to the secondary metabolites of plants, which can be classified mainly into polyphenols, terpenoids, alkaloids and sulfur-containing phytochemicals [9]. Secondary metabolites confer color, aroma, and texture and can protect plants against different injuries, such as free radicals, aggression by pathogens, or ultraviolet (UV) radiation. These metabolites are responsible for the medicinal benefits of plant extracts, which have attracted increasing interest in recent years [10,11,12,13,14]. Therefore, with the focus on natural products in the skin care sector, compounds derived from materials used in the food industry could potentially be used as antimicrobials and preservatives, as well as active compounds for the cosmetic industry, in turn, alleviating the abovementioned environmental problem.

*Prunus avium* L., a tree commonly known as SC, belongs to the Rosaceae family. SC trees are widely distributed around the world, with a higher prevalence in temperate climates. In Europe, Spain, Italy, Greece, Poland, Hungary, and Germany produce the most SC [15]. SC fruit is appreciated by consumers due to its taste, color, high content of water, and nutritional and bioactive properties as unprocessed fruit or as juice, jams, and alcoholic beverages. The antioxidant activity and phenolic composition of SC are influenced by climatic factors such as temperature, light intensity, light spectrum, and other environmental factors. These factors alter the activity of the enzyme phenylalanine ammonia-lyase, which is related to the accumulation of anthocyanins and other phenolic compounds in SC fruit [16].

The most abundant phenolic compounds in SC fruits are anthocyanins, but phenolic acids such as hydroxycinnamic acids and flavonols are also present [17,18]. In addition to fruits, SC stems are also an interesting source of bioactive compounds, and they are relatively under explored [19,20,21]. The potential of using cherries, including cherry stems, as a source of extractable bioactive compounds is high, and the use of novel and green extraction procedures, such as pressurized liquid extraction (PLE), subcritical water extraction (SWE), and supercritical fluid extraction (SFE), allows the acquisition of different compounds from SC extracts and enriches the molecular diversity in the search for new drugs and/or cosmetic ingredients.

A previous study by our group [19] provided a preliminary analysis of the compositions of SC stem extracts obtained by these techniques and serves as the basis for this study, whose aim was to study SC stems, a byproduct of SC fruit processing, and SC extracts obtained by green extractive techniques as bioactive ingredients for the cosmetic industry. Thus, the objective of this study was not only in obtaining active ingredients but also in the reutilization of a waste product from the food industry, accessing the associated economic advantages.

## 2. Materials and Methods

### 2.1. Reagents

All chemicals and reagents were of analytical reagent grade. The solvent used for extraction (ethanol) was purchased from Fisher Scientific (Madrid, Spain). For the extraction, in order to avoid possible clockage of the system, dispersive material (sea sand) and cellulose filters were acquired from Fisher Scientific (Madrid, Spain). In the analytical separation, formic acid and acetonitrile were used as mobile phase, as well as gallic acid (internal standard) were acquired from Sigma-Aldrich (Steinheim, Germany) and Fisher Scientific (Madrid, Spain) respectively. Purified water with resistance value of 18.2 MΩ for extraction and HPLC analysis was obtained from a Milli-Q system (Millipore, Bedford, MA, USA). Dulbecco’s modified Eagle’s medium (DMEM), penicillin-streptomycin, and fetal bovine serum (FBS) were obtained from Gibco (Life Technologies Co., Madrid, Spain). All other reagents were purchased from Sigma-Aldrich (Steinheim, Germany) [22,23].

### 2.2. Plant Material

*Prunus avium* stems were kindly provided by an SC producer (La Picota del Jerte, Valdastillas, Cáceres, Spain). The stems were collected in May 2015 and immediately air dried to a moisture content of 12%. Then, the stems were grounded and sieved (1 mm hole size) with a Ultra Centrifugal Mill ZM 200 (Retsch GmbH, Haan, Germany). The pulverized sample was stored avoiding light, humidity, and high temperature.

### 2.3. Green Extraction Techniques

#### 2.3.1. Pressurized Solvent Extraction (PLE)

The extraction was carried out using a Dionex ASE 350 Accelerated Solvent Extractor (Dionex Corp., Sunnyvale, CA, USA) equipped with solvent reservoirs, a pump, an oven, a cell tray, and a collection vessel tray, as described previously [19]. Briefly, 6 g of cherry stem powder was mixed with 12 g of sand and packed into a 34 mL stainless steel extraction cell. Moreover, in order to avoid possible blockage of the system by solid particles, cellulose filters and stainless steel frits were disposed at both sides of the extraction cell. The extraction was carried out with ethanol/water (1:1, *v*/*v*), at a temperature of 40 °C. The extraction was performed in static mode for 20 min at 1500 psi. The extract was collected in vials, filtered through 0.2 μm polytetrafluoroethylene (PTFE) syringe filters, and concentrated under vacuum at room temperature using a Savant SC250EXP SpeedVac Concentrator (Thermo Scientific, Waltham, MA, USA). The extracts were stored at −20 °C and protected from light exposure until use.

#### 2.3.2. Supercritical Fluid Extraction (SFE)

The SFE-CO_2_ experiments were performed as previously described using a Waters Prep Supercritical Fluid Extraction system (SFE-100) [19]. For the extraction, 5 g of SC stem powder was mixed with sea sand at a ratio of 1:2. The SFE step was carried out in dynamic mode at 40 °C using a total flow rate of 22 g/min of CO_2_ plus 15% ethanol. The extraction pressure was set at 150 bar during the 1 h process. The collected extract was concentrated in a water bath at 40 °C using a rotary evaporator, and was reconstituted in ethanol (co-solvent extraction) up to a concentration of 1000 mg/L. The extract was filtered through 0.2 μm PTFE syringe filters (Millipore, Bedford, MA, USA) and then stored at −20 °C until analysis.

#### 2.3.3. Subcritical Water Extraction (SWE)

The SWE was performed in a home-made subcritical water extractor with a 1.7 L high-pressure stainless steel vessel. The extraction was optimized as described elsewhere [21]. The sample was extracted for 30 min at a pressure of 20 bar and a temperature of 150 °C with an agitation rate of 3 Hz. The sample-to-water ratio was 1:90. A flow-through water bath at 20 °C was used to immediately cool the vessel after extraction. Then, the system was depressurized and purged with pure nitrogen. The extract was filtered, concentrated under vacuum, and stored refrigerated until analysis, as mentioned previously.

### 2.4. HPLC-ESI-QTOF-MS Analysis

The compositions of the extracts were characterized in depth using high-performance liquid chromatography coupled to electrospray quadrupole-time-of-flight mass spectrometry (HPLC-ESI-QTOF-MS). The SC stem extracts were analyzed using an Agilent 1260 HPLC instrument (Agilent Technologies, Palo Alto, CA, USA) equipped with a binary pump, an online degasser, a thermostatically controlled autosampler and column compartments, and a diode array detector. The samples were separated on an Agilent ZorBax Eclipse Plus C18 column (1.8 μm, 4.6 × 150 mm) protected by a guard cartridge packed with the same material. The mobile phases consisted of water with 0.1% formic acid as eluent A and acetonitrile as eluent B with the following elution program: at the beginning, the initial conditions were composed of 95%:5% of mobile phase A-B, at 15 min the percentages were A-B 35%:65% B, after 36 min the composition was 5%:95% of phase A-B, then the initial conditions were restored in 4 min and maintained for 5 min before the next injection. Other chromatographic parameters were 10 μL of sample injection, 0.80 mL/min flow rate, column temperature 25 °C, and sample compartment temperature 4 °C.

The mass analyzer coupled to HPLC was an Agilent 6540 Ultra High Definition (UHD) Accurate-Mass Q-TOF mass spectrometer. This detector registered the signal in negative ionization mode within a mass-to-charge ratio (*m*/*z*) range of 100–1700 *m*/*z*. The ionization of analytes were performed with a Jet Stream dual ESI interface and using pure nitrogen as nebulizer at a pressure of 20 psi. The optimized ion transfer parameters could be resumed in the use of pure nitrogen at 10 L/min and 325 °C as drying gas, and voltages of 4000 V and 130 V in the capillary and fragmentor, respectively. For a verified identification, several fragmentation analyses were carried out with different collision energies (10 eV, 20 eV, and 40 eV) in order to achieve an optimum fragmentation pattern.

Continuous infusion of the reference ions (*m*/*z* 112.985587 (trifluoroacetate anion) and 1033.988109 (adduct of hexakis(1H,1H,3H-tetrafluoropropoxy) phosphazene)) was used to correct each mass spectrum. Both reference ions provided accurate mass measurements typically better than 2 ppm.

All operations, acquisition and analysis of the data were controlled by Masshunter workstation software version B.06.00 (Agilent Technologies, Santa Clara, CA, USA).

### 2.5. Total Phenolic Content and Antioxidant Activity Assays

The three extracts from SC stems obtained by SFE, PLE, and SWE (scSFE, scPLE, and scSWE) were dissolved into ethanol EtOH, EtOH-H_2_O (50:50), and H_2_O, respectively, at the desired concentrations. All assays were evaluated in three independent analyses. The total polyphenolic content was determined by the Folin–Ciocalteu method using gallic acid as the standard (% GAE), as described previously [23]. The Trolox equivalent antioxidant capacity (TEAC) assay was performed through decoloration of the 2,2′-Azino-bis(3-ethylbenzothiazoline-6-sulfonic acid) diammonium salt ABTS radical cation (ABTS•^+^) by reducing agents as described in [22], and the results are expressed in millimole (mmol) of Trolox per 100 g of extract by dry weight. The ferric reduction antioxidant power (FRAP) was determined as described elsewhere [24,25], and the FRAP values were calculated using FeSO_4_·7H_2_O as the standard. The oxygen radical absorbance capacity (ORAC) assay was carried out on a Fluostar Galaxy spectrofluorometric analyzer (BMG Labtechnologies GmbH; Offenburg, Germany), as previously described [25], using 2,2′-azobis(2-methylpropionamidine) dihydrochloride (AAPH) as the radical generator. The ORAC values were calculated using a regression equation relating the Trolox concentration and the area under the fluorescence decay curve [22,25]. The ability of the extracts to inhibit lipid peroxidation was studied by using a thiobarbituric acid reactive substances (TBARS) assay using small unillamellar vesicles (SUVs) that were prepared by sonicating multilamellar vesicles of soybean phosphatidylcholine (Lipoid GMBH, Steinhausen, Switzerland), as described in [25].

The hydroxyl radical scavenging capacity of the extracts was determined through a modification of the ORAC method and is abbreviated as ORAC_OH_ [26,27]. In this assay, 16.7 nM β-phycoerythrin (β-PE) was used as an indicator protein, and H_2_O_2_-Cu^2+^ (0.3% H_2_O_2_, 0.3% and 9 mM CuSO_4_) was used as the hydroxyl radical generator. Quercetin (0–750 nM) was used as the control. The fluorescence of β-PE was determined every 2 min after the addition of H_2_O_2_-Cu^2+^. The areas under the β-PE decay curves were used to calculate the quercetin slope and extract slopes. The final results are expressed as micromole (µmol) of quercetin equivalents per milligram (mg) of extract.

The nitric oxide radical scavenging activity was measured using the Griess nitrite assay [28,29]. The amount of nitric oxide radical inhibition (%) was calculated using the following equation, where Abs_control_ is the absorbance of the control reaction and Abs_sample_ is the absorbance in the presence of the extract:

Inhibition % = (Abs_control_ – Abs_sample_)/Abs_control_ × 100

### 2.6. In Vitro Determination of Antiaging Properties by Enzymatic Assays

For all the enzymatic assays, scSFE was dissolved into EtOH at a final concentration of 0.02% (*w/v*), except for the hyaluronidase inhibition determination. For this assay, a concentration of 0.001% (*w/v*) scSFE was used. Vehicle was also included in controls to discard any interference. These concentrations were selected after preliminary tests to avoid color interferences from the extracts. Statistical significance was determined by comparison with the negative (untreated) control.

The inhibition of collagenase was studied through the degradation of *N*-[3-(2-furyl) acryloyl]-Leu-Gly-Pro-Ala (FALGPA), as described previously [30], and epigallocatechin gallate was used as the positive control for inhibition activity. The effect on tyrosinase was studied spectrophotometrically through the appearance of the substrate dopachrome, and kojic acid was used as the positive control, as described previously [30]. The effect on elastase was measured using *N*-succ-(Ala) 3-nitroanilide (SANA) as the substrate and phenylmethanesulfonyl fluoride (PMSF) as the positive control, as described in [30]. The hyaluronidase inhibition was determined by a method previously described [30]; the amount of *N*-acetylglucosamine after sodium hyaluronate incubation was measured, and *p*-dimethylaminobenzaldehyde was used as the positive control for inhibition. The antiglycation assay was performed with bovine serum albumin (BSA) as a substrate. Fluorescence was measured 7 days after BSA incubation with threose and diethylenetriaminepentaacetic acid, as described previously [30], using a Cytation 3 Cell Imaging Multi-Mode Reader (BioTek, Colmar Cedex, France). Aminoguanide was used as a positive control for the inhibition activity. All the samples were evaluated in each assay using three independent samples.

### 2.7. Cell Culturing

Human immortalized keratinocytes from HaCaT cell line were obtained from CLS Cell Lines Service GmbH (Eppelheim, Germany). The cells were cultured as previously described [22,23] in DMEM supplemented with 10% (*v*/*v*) FBS and 1% (*v*/*v*) of antibiotics (0.1 mg/mL penicillin and 100 U/mL streptomycin) in a humidified atmosphere with CO_2_ (5% *v*/*v*) at 37 °C. The cells were trypsinized following the manufacturer’s instructions every third day and seeded in 96-well plates (14,000 cells per well). Extracts were prepared and dissolved in dimethyl sulfoxide (DMSO) at 30 mg/mL for every assay.

### 2.8. ROS Generation and Photoprotection Measurements

The 2′,7′-dichlorodihydrofluorescein diacetate (H_2_DCF-DA) fluorescence probe (Molecular probes, Life Technologies Co., Europe) was used to determine the effect of the extract on intracellular reactive oxygen species (ROS) generation induced by UVA and UVB radiation. For this purpose, cells were cultured in black 96-well plates and maintained in medium for 24 h. The cells were washed with phosphate-buffered saline (PBS) and treated with 50 μL of PBS containing the extract (100 and 200 μg/mL, or equivalent vehicle concentration for controls) during UVB light treatment (800 or 1200 J/m^2^) or UVA radiation (3 or 6 J/m^2^) emitted from Bio-Link Crosslinker BLX-E312 and BLX-365, respectively (Vilber Lourmat, Collégien, France). After irradiation, the PBS was replaced with fresh medium, and the cells were incubated with H_2_DCF-DA (10 μg/mL) for 2 h at 37 °C and 5% CO_2_ for the oxidative stress assay. The fluorescence of H_2_DCF-DA was measured using a Cytation 3 Cell Imaging Multi-Mode Reader microplate reader (BioTek, Colmar Cedex, France) with 495 nm excitation and 520 nm emission filters.

The inhibition of ROS was calculated as follows:

Inhibition of ROS (%) = 100 × (C_UV_ – sample)/(C_UV_ – 100)

All parameters represent the ROS values normalized to the appropriate nonirradiated controls. C_UV_ is the fluorescence signal of the irradiated control without treatment, and sample is the fluorescence signal for the extract-treated samples at the desired UV dose. The results are reported as the mean ± SD of six determinations.

In addition, after washing with PBS, the 3-(4,5-dimethylthiazol-2-yl)-2,5-diphenyltetrazolium (MTT) assay was used to determine cell viability after 24 h of UVB irradiation to determine the photoprotective effect of the extracts [14,22,23]. The photoprotection was calculated as follows:

Photoprotection (%) = 100 × (C_UV_ – sample)/(C_UV_ – 100)

where C_UV_ is the signal of the irradiated control without treatment and sample is the signal of the extract-treated sample at the corresponding UVB dose. The results are expressed as the mean ± SD of six determinations.

### 2.9. Statistical Analysis

Statistical comparisons were developed using GraphPad Prism software v6.0 (GraphPad Software, San Diego, CA, USA). One-way ANOVA and Tukey’s post-test were employed for data analysis. Differences were considered statistically significant when *p* < 0.05, detailed in the figures using the following symbols: * *p* < 0.05, ** *p* < 0.01, *** *p* < 0.001, and **** *p* < 0.0001 [22,23].

## 3. Results and Discussion

### 3.1. Characterization of Sweet Cherry Stem Extracts by HPLC-ESI-QTOF-MS

**Figure 1:**
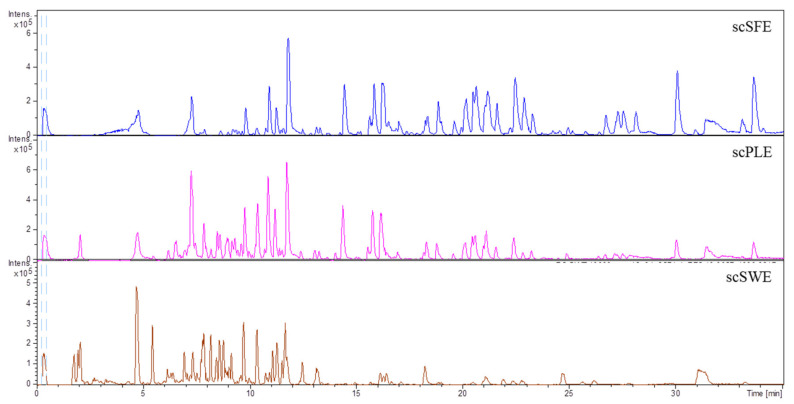
Base peak chromatograms of the extracts from SC stems obtained by supercritical fluid extraction (SFE; scSFE), pressurized solvent extraction (PLE; scPLE), and subcritical water extraction (SWE; scSWE).

The scPLE, scSFE, and scSWE extracts were fully characterized by HPLC-ESI-QTOF-MS. For this analysis, the dried extracts were reconstituted in EtOH, EtOH-H_2_O (50:50), and H_2_O, respectively, up to a concentration of 1000 mg/L. Their chromatographs are shown in Figure 1.

The compounds were tentatively identified using the information provided by the software (accurate masses, isotopic distributions, MS spectra, and molecular formula), together with the fragmentation patterns obtained from tandem mass spectrometry (MS/MS) experiments in comparison with standards when available or data previously reported in the literature. A total of 57 compounds were identified from 4 different families: (1) organic acids, phenolic acids, and derivatives (8 compounds); (2) flavonoids and derivatives (36 compounds); (3) fatty acid derivatives (9 compounds); and (4) terpenes (4 compounds). Thus, 18 of these compounds are herein identified for the first time in this matrix. The identities of the obtained compounds are summarized in Table 1, and these results significantly advance and complete the previous data available on these extracts obtained using both gas chromatography coupled to mass spectrometry (GC-MS) [21] and HPLC-ESI-QTOF-MS [19].

**Figure 2:**
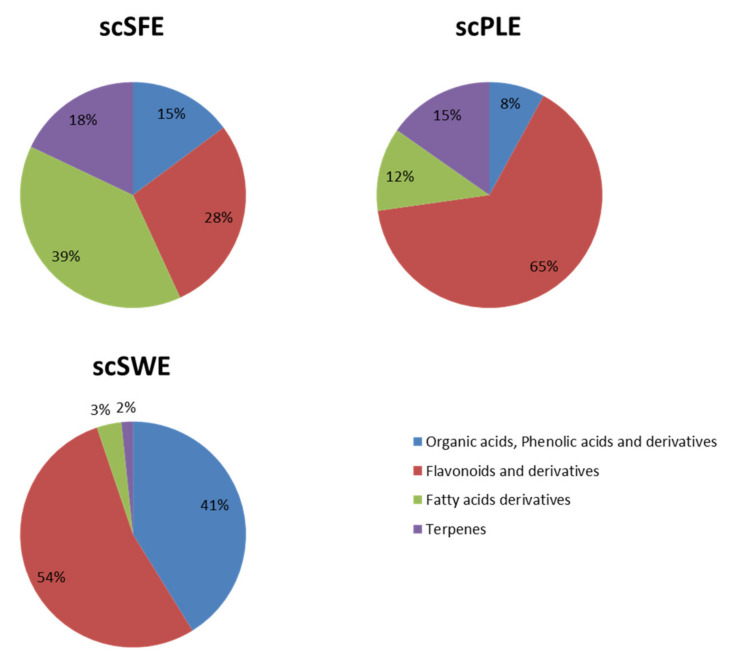
Semiquantitative data regarding the different families of compounds extracted by SFE (scSFE), PLE (scPLE), and SWE (scSWE).

Semiquantitative comparisons among the different extraction techniques regarding the presence of individual compounds in those extracts can be observed in Table 2. This information is useful for determining which technique is better for extracting particular types of compounds. In general, as depicted in Figure 2, organic and phenolic acids and derivatives are present at higher concentrations in the extract obtained by SWE (scSWE). In addition, flavonoids were more abundant in the PLE extract (scPLE); as expected, fatty acid derivatives and terpenes, which are nonpolar in nature, were better extracted by SFE (scSFE). Similar results have been reported for other natural compounds, such as marine compounds [31] and polyphenols [32].

### 3.2. Total Phenolic Contents and Antioxidant Capacities of the SC Stem Extracts

After the analytical processing of the extracts, a multistep screening of the three extracts was conducted in three stages to select the best extract with the greatest potential as a novel cosmetic ingredient (see graphical abstract). This screening was designed to evaluate the most relevant biological activities for the cosmetic industry using a collection of in vitro and cellular assays, all of which are described in the Materials and Methods section.

The first step evaluated the total phenolic content (TPC). This assay was the first step because polyphenolic compounds are one of the main groups of compounds known to show anti-aging effects and present other biological activities [33,34]. Furthermore, higher polyphenolic contents typically result in more intense biological activities [35,36]. The TPC was evaluated using the Folin-Ciocalteu assay, as described in the Materials and Methods Section. In addition to TPC measurement, this first stage included the TEAC assay, which is accepted as a general method for measuring antioxidant activity. The relationship between the total polyphenolic content and the antioxidant activity has been demonstrated previously by numerous studies published by our group [25,37,38] and others [35,36].

The results for this first stage are presented in Table 3. Among the SC stem extracts, scPLE and scSFE had the highest total polyphenolic contents, and their contents were not significantly different (*p* > 0.05). scSFE showed the highest antioxidant effect in the TEAC assay, followed by scPLE. However, scSWE showed a remarkably lower TPC and antioxidant capacity (at least *p* < 0.001 and *p* < 0.0001, respectively). Thus, both assays showed similar trends for the three extracts, with the lowest antioxidant activity for that extract with the lowest polyphenolic content, as expected. On the basis of these results, scSWE was not included in for further screening steps due to its poorer results.

In a second stage, additional antioxidant assays, FRAP and ORAC, were carried out to clarify the antioxidant activities of the selected extracts (scSFE and scPLE). FRAP estimates the Fe(III) reducing activity, whereas the ORAC assay determines the activity related to chain-breaking antioxidants, which is directly related to peroxyl radicals. These analyses are more closely related to the biological function of antioxidants [25,39]. The results are shown in Table 4. Both extracts presented significant antioxidant activities with higher values for scSFE in the ORAC assay and for scPLE in the FRAP assay. These values are higher than those of other extracts from *Cistus* sp. plants obtained by aqueous and hydroalcoholic conventional extraction methods previously characterized by our group [25,38].

Although scSFE and scPLE presented some differences, probably due to differences in their composition, as shown in Section 3.1, both exhibited high antioxidant activities that deserve further investigation. The higher potency of scPLE compared with scSFE in the FRAP assay was probably due to scPLE’s higher content of flavonoids bearing a catechol group in their B ring, such as catechins and quercetin derivatives, which can complex metal ions. However, the results for scSFE were more interesting from a cosmetic point of view, as ORAC indicates the capacity to scavenge peroxyl free radicals and other radicals derived from lipid peroxidation, and these radicals are frequent in cosmetic products due to the inclusion of oily ingredients in their formulation. Furthermore, the SFE extraction technique is more suitable for scale-up for use at large industrial facilities than is PLE, which is a less developed extraction technique at the industrial level. For these reasons, scSFE was selected for full characterization in the further assays included in the third stage of the screening.

The third stage further elucidated the antioxidant capacity of scSFE through additional antioxidant assays. In this sense, TBARS for the specific study of lipid peroxidation, a modified ORAC method based on OH^·^ radicals, and a Griess nitrite-based assay for nitric oxide radicals were performed. The results for all these assays are shown in Table 5.

scSFE showed a significant antioxidant capacity in all the assays, suggesting that this extract is a good candidate for use as a bioactive ingredient against oxidative stress, as the extract has shown antioxidant capacity through different methods and against different targets, such as lipid peroxidation and different kinds of free radicals.

Antioxidant activities are highly desirable for cosmetic ingredients for several reasons. On the one hand, this activity protects the final formula itself from oxidation, especially from oxidation related to its oily ingredients. In addition to this advantage, which is mainly related to the final product formulation, the antioxidant activity of the ingredients is probably one of the most commonly used claims in cosmetic products. In this sense, natural extracts have been shown to reduce oxidative stress, mainly due to their polyphenols [40,41]. scSFE contains different families of compounds, as shown in Figure 2, and polyphenols (including flavonoids, organic acids, phenolic acids, and their derivatives) were the most abundant. Thus, the antioxidant effects of scSFE could be mainly due to the polyphenolic compounds present in this extract, but contributions from other compounds, especially terpenes, cannot be discarded. Catechins, naringenin, and chrysin are the main polyphenols in this extract, and thus the reduction in lipid peroxidation and the depletion of hydroxyl radicals and nitric oxide could have been due to these compounds, which have been demonstrated to have antioxidant capacities. Naringenin is a flavanone, catechin is a flavanol, and chrisin is a flavone. Compounds of these types have been shown to have antioxidant activities. Naringenin, which is found in citrus fruits, grapes, and other fruits, has shown antioxidant effects through lipid peroxidation reduction; increases in antioxidant defense; and scavenging free radicals, such as hydroxyl, superoxide, hydrogen peroxide, and nitric oxide radicals [42,43]. Moreover, catechins have been shown to have antioxidant activity through many assays, such as the ABTS and FRAP assays. They protect against AAPH-induced peroxide radicals and lipid peroxidation and can scavenge free radicals [44,45]. In addition to naringenin and catechins, chrysin and its derivatives reduce lipid peroxidation, regulate redox homeostasis, and increase antioxidant enzymes [46,47]. The antioxidant effects of these compounds are related to the carbonyl group at C-4 and the double bond between C2 and C3 [48].

### 3.3. Skin Aging-Related Enzymatic Assays

In this third stage, the putative modulative activities of scSFE on some of the most relevant enzymes related to skin health and appearance were also tested. In this sense, the activities of collagenase, elastase, hyaluronidase, and tyrosinase were challenged with scSFE, as detailed in the Materials and Methods section. These experiments were concluded with a study of the inhibition of advanced glycosylation end product (AGE) formation, conducted as described in the Materials and Methods section. The inhibition of collagenase, tyrosinase, elastase, hyaluronidase, and AGE formation are related to the prevention of the degradation of extracellular matrix (ECM), skin preservation, and antiaging. In fact, plant extracts have been shown to inhibit tyrosinase, collagenase, elastase, and hyaluronidase activity [49,50,51]. The results, shown in Table 6, are expressed as the percentage of inhibition for each assay.

Collagen plays a critical role in the appearance and function of the skin; it confers tensile strength and resiliency to the skin and is the main protein in the ECM of the dermis. Its degradation is related to skin wrinkling and aging. Collagenase inhibition is related to the maintenance of skin tensile strength and elasticity, even more so in collagenase induction by ROS or irradiation, which are important factors in aging. In this case, scSFE did not present any collagenase inhibition activity, presenting a negative value, which means that its effect was weaker than that of the negative control but was not statistically significant.

Tyrosinase is an enzyme involved in melanin production, the main defense of organisms against UV irradiation. Melanin absorbs UV radiation and reduces the formation of photoproducts that could be harmful to the skin [52]. Tyrosinase induction is related to skin protection through an increase in melanin production, and tyrosinase inhibition could be useful in diseases such as vitiligo. As shown in Table 6, scSFE showed a moderate but not statistically significant effect compared with the untreated negative control.

Elastin is an extracellular matrix protein responsible for elasticity in the dermis and other connective tissues by forming elastin fibers. Elastase is an enzyme able to degrade elastin, leading to skin aging and wrinkles. Therefore, the inhibition of elastase is related to skin aging and wrinkle protection. The results in Table 6 indicate that scSFE showed potent elastase inhibition activity, even above the PMSF positive control, and that the effect was significant (** *p* < 0.01).

Hyaluronic acid is found in connective tissue and is part of the ECM. Hyaluronic acid presents water holding properties and maintains the viscosity and the correct permeability of connective tissues and maintains skin hydration. Hyaluronidase degrades hyaluronic acid, and its inhibition is related to the maintenance of high levels of hyaluronic acid, improving the general aspect of skin and specifically skin hydration. scSFE presented potent hyaluronidase inhibition activity, reaching almost 100% of the level obtained for *p*-dimethylaminobenzaldehyde (positive control), and the effect was highly significant (**** *p* < 0.0001).

Finally, oxidative stress increases protein glycation, which is responsible for advanced glycosylation end products (AGEs) in skin. AGEs are one of the causes of collagen degradation, leading to skin aging. The inhibition of protein glycation is related to the prevention of aging and wrinkling. As expected by its antioxidant capacity shown in the previous section, scSFE was able to reduce AGE formation by 50% with high statistical significance (**** *p* < 0.0001).

A wide variety of phytomolecules belonging to different classes of polyphenols, terpenoids, or steroids (e.g., catechins, carnosic acid, ellagic acid, curcumin, and hydroxycinnamic acids) are inhibitors of collagenase, elastase, and hyaluronidase [53,54,55,56]. Some plant extracts containing these compounds scavenge free radicals, mainly due to polyphenols, protecting the skin matrix through the inhibition of enzymatic degradation and/or promoting the synthesis of its components, improving skin elasticity and tightness [57,58]. The polyphenols present in scSFE could be responsible for its ability to inhibit cosmetic enzymes. As shown in [59], catechin and epigallocatechin gallate inhibit collagenase and elastase, and naringenin inhibits hyaluronidase. This activity is related to the number of hydroxyl groups, as more available hydroxyl groups result in higher activity, and the inhibition of these enzymes decreases with substitution of hydroxyl groups or glycosylation [60]. Furthermore, an extract of *Libidibia ferrea*, whose main constituents are ellagic acid, catechin, and epicatechin, inhibited elastase, hyaluronidase, and tyrosinase, but presented a weak inhibition of collagenase, similar to what is seen with scSFE [61]. These results may suggest that catechins, which are the main components of scSFE, could be responsible for the activity observed in these cosmetic assays. Further studies must be conducted to identify the molecules related to each inhibition activity, as well as their inhibition mechanisms, as it is documented that natural compounds can interact with these enzymes through different methods, such as competitive and/or noncompetitive inhibition [59,62].

The overproduction or accumulation of melanin could lead to pigmentary disorders such as vitiligo, and it is related to skin aging and photoprotection. Tyrosinase is the enzyme that regulates the hydroxylation of L-tyrosine to form 3,4-Dihydroxy-L-phenylalanine (L-DOPA), a precursor of melanin. Inhibiting tyrosinase is a method for avoiding disorders related to skin hyperpigmentation, and in vitro enzymatic assays, as employed in the present study, are significantly related to melanin synthesis in melanocytes [63]. Some polyphenols obtained from plants can inhibit tyrosinase and melanogenesis. In fact, catechin and its derivates, such as those present in scSFE, potently inhibit tyrosinase, and thus these flavanols could be the main flavanols responsible for the tyrosinase inhibition shown by scSFE. In addition, some polyphenol mixtures, such as mixtures of glabridin and resveratrol, show a synergistic tyrosinase inhibition [64]. A synergistic effect could increase the value of plant extracts such as scSFE, which are characterized by the presence of many different compounds. However, a synergistic approach similar to that described in [65] must be developed after the identification of the products responsible for the tyrosinase inhibition and other biological activities.

### 3.4. scSFE Showed Photoprotection Activity against UVB Irradiation

**Figure 3:**
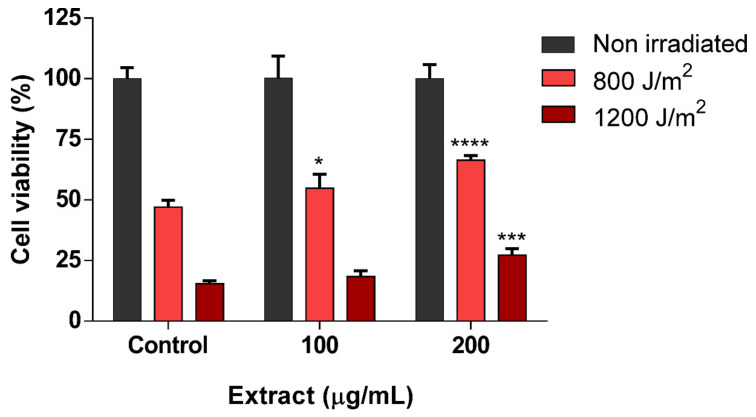
Effect of scSFE extract (100 or 200 µg/mL) on viability after UVB (800 and 1200 J/m^2^) irradiation. Data are expressed as the mean of six replicates ± SD. * (*p* < 0.05), *** (*p* < 0.001), and **** (*p* < 0.0001) indicate statistically significant differences compared to an irradiated sample in the absence of the extract. Each condition is normalized with respect to its non-irradiated control.

As the last set in the third stage of the screening, the photoprotective effect of scSFE was evaluated in HaCaT cells. Viability after UVB irradiation (800 or 1200 J/m^2^) was first determined through MTT assay in the presence of different concentrations of scSFE (Figure 3).

scSFE extract increased the viability of cells 24 h after UVB irradiation compared to the irradiated control. At 800 J/m^2^, 100 µg/mL scSFE extract (0.01% *w/v*) increased cell viability compared to untreated irradiated cells, whereas 100 µg/mL extract did not protect against the 1200 J/m^2^ dose. This protective effect was greater in the highest treatment concentration (200 µg/mL, 0.02% *w/v*) after 800 and 1200 J/m^2^ irradiation, with a statistically significant protective effect compared to the untreated control. The highest photoprotection activities were observed for 800 J/m^2^, 14.61% with 100 µg/mL extract, and 36.53% photoprotection with 200 µg/mL extract. However, weaker effects were obtained after 1200 J/m^2^ of UVB irradiation, with 3.51% and 13.99% photoprotection, respectively, compared to control as shown in Table S1. Similar results with other natural extracts, such as citrus, rosemary, and lemon balm extracts, have been obtained using the same technique, with protection levels ranging from 10% to 80% [22,23,66].

This photoprotective activity of scSFE could be due to various factors. On the one hand, scSFE showed a significant absorption in the UV range in a dose-dependent manner, and thus a substantial portion of the observed keratinocyte photoprotection could be due to the ability of the compounds present in this extract to absorb and scavenge UVB radiation, as many plant extracts have been shown to do [23,67,68,69]. On the other hand, intracellular mechanisms may be involved in scavenging UVB-induced free radicals, attenuating death mechanisms and/or DNA damage, as other plant extracts have been shown to do [23,70,71,72]. In fact, some of the main compounds present in scSFE have photoprotective activities through different mechanisms. Naringenin has been shown to increase keratinocyte survival and inhibit apoptosis and pyrimidine dimers after UVB radiation [73]. Epigallocatechin gallate reduces UVB-induced damage in keratinocytes [74,75], and catechin may protect skin cells against UVB-induced damage through its antioxidant activity [76]. In addition, chrysin has shown UVB protection activity by attenuating UVB-induced apoptosis, ROS generation, and cyclooxygenase 2 expression [77,78]. All these data suggest that the photoprotective effects of scSFE could be due to the different activities of the polyphenols naringenin, catechin, chrysin, and their derivates, which are the main polyphenolic compounds present in scSFE.

### 3.5. scSFE Inhibited Intracellular ROS Generation Induced by UVA and UVB Light in HaCaT Cells

**Figure 4:**
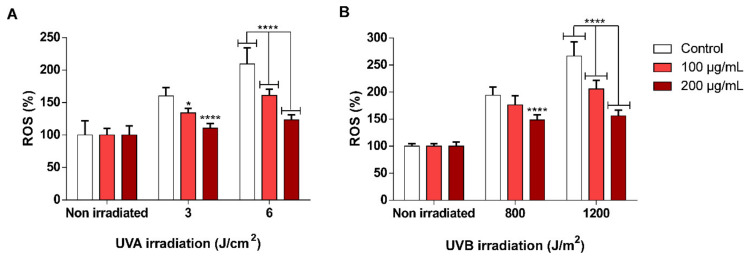
Determination of the antioxidant effects of scSFE related to UVA- (**A**) and UVB- (**B**) induced reactive oxygen species (ROS) generation in HaCaT cells. Fluorescence was normalized to non-irradiated controls. The white bars indicate the fluorescence signal in the absence of treatment for each condition. The data are expressed as the mean ± SD (*n* = 6). * (*p* < 0.05) and **** (*p* < 0.0001) indicate significant differences compared with irradiated cells at the same UVA dose (3 or 6 J/cm^2^) or UVB dose (800 or 1200 J/m^2^) in the absence of scSFE.

As mentioned in the previous section, one of the putative mechanisms involved in scSFE photoprotection may be its antioxidant properties. To check if the antioxidant properties shown by scSFE in the previous sections were also present at the cellular level, the antioxidant capacity of the extract was evaluated in vitro through the determination of intracellular ROS in the HaCaT cell line. Ultraviolet (UV) A and B were used to induce oxidative stress, as determined by measuring dichlorofluorescein-diacetate H_2_DCF-DA fluorescence, as described in the methods section. Figure 4 shows ROS generation after UVA (3 or 6 J/cm^2^) or UVB (800 or 1200 J/m^2^) irradiation in the absence or presence of the extract compared to the control (without irradiation).

Oxidative stress was inhibited by all the concentrations at all the UV doses (both UVA and UVB) tested in this assay. The reduction of fluorescence observed in all conditions is displayed in Table S2.

H_2_DCF-DA is a fluorescent probe that is particularly sensitive to H_2_O_2_, ^•^OH, and peroxynitrite radicals at the intracellular level [79]. The ORAC_OH_ assay showed that scSFE has a significant capacity to scavenge ^•^OH, a harmful radical that can be derived from the Fenton reaction of H_2_O_2_ or from lipid peroxides, and a significant capacity to eliminate NO^•^ radicals, which may form peroxynitrite upon reaction with O_2_^•^^−^. Therefore, the photoprotective properties of scSFE shown in this study may be related to its capacity to decrease the generation of intracellular radical species such as H_2_O_2_, ^•^OH, or peroxynitrite, which can damage a wide range of molecules in cells, including proteins and DNA [23,80]. The major polyphenolic compounds in scSFE, catechin, chrysin, and naringenin have previously shown antioxidant activity against these free radicals [42,43,44,45,46,47], with concomitant antioxidant activity against UV-induced oxidative stress [44,73,78], confirming these statements. Similar antioxidant and photoprotective effects from UVA and UVB radiations have been also documented for a well-known cosmetic ingredient, ascorbic acid [81,82,83], reinforcing the putative potential of scSFE extract as a new cosmetic ingredient.

## 4. Conclusions

New cosmetic ingredient development is a long and costly process, but there is continuous demand for new products in the cosmetic market. This manuscript tries to address this need through the revalorization of agricultural byproducts such as SC stems.

According to the obtained results, scSFE is a strong candidate for use as a new cosmetic ingredient, especially due to its antioxidant properties, especially against lipid peroxidation, its activity against skin aging-related enzymes, and its photoprotective capability. As indicated in previous studies, these actions are related its main polyphenolic compounds—catechin, chrysin, and naringenin. However, further studies must be performed on three main topics: the molecular mechanisms involved in these biological activities, the putative pharmacological interactions between the scSFE main compounds, and the compatibility and stability of these compounds or the whole extract when incorporated in a final cosmetic formula.

## Figures and Tables

**Table 1:** Analytical data obtained from high-performance liquid chromatography coupled to electrospray quadrupole-time-of-flight mass spectrometry (HPLC-ESI-QTOF-MS) analysis for SC extracts.

Retention Time (min)	*m*/*z* Experimental	*m*/*z* Calculated	(M – H)^–^	Error (ppm)	Proposed Compound	Extracts
1.91	195.0499	195.0510	C_6_H_11_O_7_	5.7	D-gluconic acid	scSWE
1.99	191.0552	191.0561	C_7_H_11_O_6_	5.0	quinic acid	scSWE
5.42	315.0702	315.0722	C_13_H_15_O_9_	6.3	protocatechuic acid hexoside	scSWE
6.52	577.1381	577.1351	C_30_H_25_O_12_	−5	proanthocyanidin B2 isomer 1	scSFE
6.81	341.0856	341.0878	C_15_H_17_O_9_	6.6	caffeic acid hexoside	scSFE, scSWE
7.23	289.0732	289.0718	C_15_H_13_O_6_	−5.1	(epi)catechin isomer 1	scSFE, scPLE, scSWE
7.31	401.1446	401.1453	C_18_H_25_O_10_	1.7	benzyl β-primeveroside	scSWE
7.40	137.0243	137.0244	C_7_H_5_O_3_	0.6	salicylic acid	scSWE
7.43	577.1381	577.1351	C_30_H_25_O_12_	−5.1	proanthocyanidin B2 isomer 2	scSFE
7.54	771.1977	771.1989	C_33_H_39_O_21_	1.6	quercetin-rutinoside-glucoside	scSWE
7.60	521.2002	521.2028	C_26_H_33_O_11_	5.1	dihydrodehydrodiconiferyl alcohol glucopyranoside	scSWE
7.64	325.0930	325.0929	C_15_H_17_O_8_	−0.3	*p*-coumaric acid *O*-hexoside	scSFE, scPLE
7.74	449.1094	449.1089	C_21_H_21_O_11_	−1.1	eriodictyol glucoside isomer 1	scSWE
7.81	165.0557	165.0557	C_15_H_17_O_8_	0.4	melilotic acid	scSWE
7.83	289.0735	289.0718	C_15_H_13_O_6_	−5.9	(epi)catechin isomer 2	scSFE, scPLE
7.88	449.1094	449.1089	C_21_H_21_O_11_	−1.1	eriodictyol glucoside isomer 2	scSWE
8.16	195.0664	195.0663	C_10_H_11_O_4_	−0.8	dihydroferulic acid	scSWE
8.45	609.1482	609.1461	C_27_H_29_O_16_	−2.1	rutin	scSFE, scSWE
8.58	465.1073	465.1038	C_21_H_21_O_12_	−3.4	epicatechin-*O*-glucuronide	scSFE, scPLE, scSWE
8.85	463.0903	463.0882	C_21_H_19_O_12_	−2.1	quercetin-glucoside	scPLE, scSWE
8.91	431.1011	431.0984	C_21_H_19_O_10_	−6.2	genistein-*O*-glucoside isomer 1	scSFE, scPLE, scSWE
8.95	593.1534	593.1512	C_27_H_29_O_15_	−3.7	kaempferol-*O*-rutinoside	scSFE, scPLE, scSWE
9.13	477.1076	477.1038	C_22_H_21_O_12_	−7.9	isorhamnetin-glucoside	scSFE, scPLE
9.15	431.1007	431.0984	C_21_H_19_O_10_	−5.3	genistein-*O*-glucoside isomer 2	scSFE, scPLE
9.28	431.1004	431.0984	C_21_H_19_O_10_	−4.6	genistein-*O*-glucoside isomer 3	scSFE, scPLE, scSWE
9.42	447.0955	447.0933	C_21_H_19_O_11_	−4.9	kaempferol-*O*-glucoside	scSFE, scPLE, scSWE
9.58	431.0995	431.0984	C_21_H_19_O_10_	−2.7	genistein-*O*-glucoside isomer 4	scSFE, scPLE
9.75	433.1158	433.1140	C_21_H_21_O_10_	−4.1	naringenin-*O*-glucoside isomer 1	scSFE, scPLE, scSWE
10.25	433.1123	433.1140	C_21_H_21_O_10_	4.0	naringenin-*O*-glucoside isomer 2	scSFE, scPLE
10.35	417.1182	417.1191	C_21_H_21_O_9_	2.2	liquiritin	scSWE
10.69	433.1148	433.1140	C_21_H_21_O_10_	−1.7	naringenin-*O*-glucoside isomer 3	scSFE, scPLE
10.74	447.1295	447.1297	C_22_H_23_O_10_	0.3	sakuranin	scSWE
10.84	415.1064	415.1035	C_21_H_19_O_9_	−6.2	chrysin-*O*-glucoside	scSFE, scPLE
10.94	447.129	447.1297	C_22_H_23_O_10_	1.6	sakuranetin glucopyranoside	scSWE
11.07	417.1204	417.1191	C_21_H_21_O_9_	−3.1	sakuranetin xylopyranoside	scSWE
11.17	433.1176	433.1140	C_21_H_21_O_10_	−8.2	naringenin-*O*-glucoside isomer 4	scSFE, scPLE
11.27	417.1196	417.1191	C_21_H_21_O_9_	−1.2	prupersin B	scSWE
11.62	447.1307	447.1297	C_22_H_23_O_10_	−2.4	dihydrowogonin glucoside	scSWE
12.39	327.2175	327.2177	C_18_H_31_O_5_	0.6	trihydroxy-octadecadienoic acid	scSFE, scPLE, scSWE
12.78	433.1161	433.1140	C_21_H_21_O_10_	−4.9	naringenin-*O*-glucoside isomer 5	scSFE, scPLE
13.04	329.2346	329.2333	C_18_H_33_O_5_	−3.8	trihydroxy-octadecenoic acid	scSFE, scPLE, scSWE
13.23	271.0607	271.0612	C_15_H_11_O_5_	1.7	naringenin isomer 1	scSFE, scPLE, scSWE
13.53	271.0600	271.0612	C_15_H_11_O_5_	4.2	naringenin isomer 2	scSFE, scPLE
14.35	517.3187	517.3171	C_30_H_45_O_7_	−3.2	jaligonic acid	scSFE, scPLE
15.74	501.3250	501.3222	C_30_H_45_O_6_	−5.7	hydroxyceanothic acid isomer 1	scSFE, scPLE
16.14	253.0503	253.0506	C_15_H_9_O_4_	1.4	chrysin	scSFE, scPLE, scSWE
16.31	285.0775	285.0768	C_16_H_13_O_5_	−2.3	methylnaringenin	scSFE, scPLE, scSWE
16.44	255.0644	255.0663	C_15_H_11_O_4_	7.3	pinocembrin	scSFE, scPLE, scSWE
16.77	501.3238	501.3222	C_30_H_45_O_6_	−3.2	hydroxyceanothic acid isomer 2	scSFE, scPLE
19.53	293.2122	293.2122	C_18_H_29_O_3_	0.0	hydroxy-octadecatrienoic acid isomer 1	scSFE, scPLE
19.87	293.2104	293.2122	C_18_H_29_O_3_	6.1	hydroxy-octadecatrienoic acid isomer 2	scSFE, scPLE
21.52	295.2291	295.2279	C_18_H_31_O_3_	−4.2	hydroxy-octadecadienoic acid	scSFE, scPLE
23.21	293.2111	293.2122	C_18_H_29_O_3_	3.7	hydroxy-octadecatrienoic acid isomer 3	scSFE, scPLE
30.02	277.2182	277.2173	C_18_H_29_O_2_	−3.4	linolenic acid	scSFE, scPLE
31.41	455.3528	455.3531	C_30_H_47_O_3_	0.7	ursolic acid	scSWE
33.64	279.2327	279.2330	C_18_H_31_O_2_	1.1	linoleic acid	scSFE, scPLE
34.06	299.2590	299.2592	C_18_H_35_O_3_	0.4	hydroxy-octadecanoic acid	scSFE, scPLE

**Table 2:** Relative peak areas of the identified compounds in SC stem extracts expressed as mean ± standard deviation of the three analyses replicates (ND: non-detected compound). For each compound, the best extractive technique is marked in bold format. PLE: pressurized liquid extraction, SWE: subcritical water extraction, SFE: supercritical fluid extraction.

Proposed Compound	Peak Area x E+4		
	PLE	SFE	SWE
**Organic acids, phenolic acids, and derivatives**			
D-gluconic acid	ND	ND	**22.8 ± 0.9**
quinic acid	ND	ND	**32.0 ± 2.0**
caffeic acid hexoside	**5.0 ± 0.3**	ND	2.2 ± 0.2
*p*-coumaric acid *O*-hexoside	**12.0 ± 2.0**	4.0 ± 0.2	ND
protocatechuic acid hexoside	ND	ND	**17.2 ± 0.2**
salicylic acid	ND	ND	**9.9 ± 0.5**
melilotic acid	ND	ND	**16.6 ± 0.7**
dihydroferulic acid	ND	ND	**22 ± 2**
**Flavonoids and derivatives**			
(epi)catechin–(epi)catechin (proanthocyanidin B2) isomer 1	**129.0 ± 8.0**	ND	ND
(epi)catechin isomer 1	**573.0 ± 14.0**	282.0 ± 23.0	5.5 ± 0.3
(epi)catechin–(epi)catechin (proanthocyanidin B2) isomer 2	**112.0 ± 9.0**	ND	ND
(epi)catechin isomer 2	**214.0 ± 44.0**	44 ± 4	ND
rutin	**121.0 ± 3.0**	ND	13.4 ± 0.4
epicatechin-O-glucuronide	**113.0 ± 1.0**	20.0 ± 0.5	25.0 ± 1.0
quercetin-glucoside	**67.0 ± 0.6**	ND	11.0 ± 1.0
genistein-O-glucoside isomer 1	**89.0 ± 0.1**	12.0 ± 0.5	27.8 ± 0.9
kaempferol-O-rutinoside	**91.0 ± 4.0**	4.0 ± 0.2	6.3 ± 0.5
isorhamnetin-glucoside	**15.0 ± 0.2**	3.0 ± 0.3	ND
genistein-O-glucoside isomer 2	**65.0 ± 4.0**	16.0 ± 1.0	ND
genistein-O-glucoside isomer 3	**102 ± 0.8**	24.0 ± 1.0	8.4 ± 0.3
kaempferol-O-glucoside	**41.0 ± 2.0**	8.0 ± 0.2	4.6 ± 0.6
genistein-O-glucoside isomer 4	**82.0 ± 0.0**	17.0 ± 1.0	ND
naringenin-O-glucoside isomer 1	**94.0 ± 0.2**	34.0 ± 1.0	38 ± 1
naringenin-O-glucoside isomer 2	**89.0 ± 0.4**	40.0 ± 1.0	ND
naringenin-O-glucoside isomer 3	**57.0 ± 1.0**	34.0 ± 4.0	ND
chrysin-O-glucoside	**432.0 ± 14.0**	180.0 ± 1.0	ND
naringenin-O-glucoside isomer 4	**91.0 ± 0.1**	49.0 ± 5.0	ND
naringenin-O-glucoside isomer 5	6.0 ± 0.0	**10.0 ± 0.5**	ND
naringenin isomer 1	**14.0 ± 1.0**	13.0 ± 0.8	8.8 ± 0.2
naringenin isomer 2	**9 ± 0.2**	5 ± 0.4	ND
chrysin	143.0 ± 12.0	**143 ± 3**	3.8 ± 0.8
methylnaringenin	39.0 ± 2.0	**42.0 ± 1.0**	3.0 ± 0.3
benzyl β-primeveroside	ND	ND	**24.6 ± 0.4**
quercetin-rutinoside-glucoside	ND	ND	**5.9 ± 0.6**
dihydrodehydrodiconiferyl alcohol glucopyranoside	ND	ND	**4.6 ± 0.3**
eriodictyol-glucoside isomer 1	ND	ND	**14.4 ± 0.6**
eriodictyol-glucoside isomer 2	ND	ND	**36 ± 1**
liquiritin	ND	ND	**24 ± 1**
sakuranin	ND	ND	**4.3 ± 0.3**
sakuranetin-glucopyranoside	ND	ND	**4.9 ± 0.3**
sakuranin-xylopyranoside	ND	ND	**18.2 ± 0.2**
prupersin B	ND	ND	**22.3 ± 0.4**
dihydrowogonin glucoside	ND	ND	**35 ± 2**
pinocembrin	ND	ND	**5.3 ± 0.1**
**Fatty acid derivatives**			
trihydroxy-octadecadienoic acid	**42.0 ± 6.0**	29.0 ± 2.0	11.6 ± 0.6
trihydroxy-octadecenoic acid	**48.0 ± 2.0**	40.0 ± 2.0	7.4 ± 0.2
hydroxy-octadecatrienoic acid isomer 1	19.0 ± 0.3	**59.0 ± 0.2**	ND
hydroxy-octadecatrienoic acid isomer 2	9.0 ± 0.5	**40 ± 0.8**	ND
hydroxy-octadecadienoic acid	76.0 ± 2.0	**210.0 ± 12.0**	ND
hydroxy-octadecatrienoic acid isomer 3	57.0 ± 4.0	**121.0 ± 5.0**	ND
linolenic acid	129.0 ± 15.0	**407.0 ± 39.0**	ND
linoleic acid	129.0 ± 2.0	**448.0 ± 39.0**	ND
hydroxy-octadecanoic acid	13.0 ± 0.3	**49.0 ± 3.0**	ND
**Terpenes**			
jaligonic acid	**333.0 ± 2.0**	297.0 ± 10.0	ND
ursolic acid	ND	ND	**58.0 ± 11.0**
hydroxyceanothic acid isomer 1	**279.0 ± 7.0**	273.0 ± 6.0	ND
hydroxyceanothic acid isomer 2	54.0 ± 3.0	**78.0 ± 6.0**	ND

**Table 3:** Percentage of gallic acid equivalents (GAE) ± standard deviation (SD) determined by the Folin-Ciocalteu assay and antioxidant capacity in millimole (mmol) Trolox eq./100 g extract ± SD, determined through Trolox equivalent antioxidant capacity (TEAC) for scSFE, scPLE, and scSWE.

Extract	Folin % GAE (w/w)	TEAC mmol Trolox eq./100 g Extract
scSFE	15.26 ± 2.94	240.61 ± 11.74
scPLE	18.81 ± 2.36	220.53 ± 13.57
scSWE	5.49 ± 1.16	70.38 ± 3.89

**Table 4:** Antioxidant capacity of scSFE and scPLE by different methods: ferric reduction antioxidant power (FRAP) and oxygen radical absorbance capacity (ORAC), giving the antioxidant capacities compared to FeSO_4_ and Trolox, respectively. All the results are presented as the mean ± SD.

Extract	FRAP mmol FeSO_4_ eq./100 g Extract	ORAC mmol Trolox eq./100 g Extract
scSFE	64.83 ± 6.32	107.77 ± 5.76
scPLE	203.94 ± 8.37	64.15 ± 1.04

**Table 5:** Antioxidant capacity of scSFE. The results showed its activity against lipid peroxidation through a thiobarbituric acid reactive substances (TBARS) assay (% inhibition of lipid peroxidation of 5 mg/mL of extract), the hydroxyl radical capacity as determined by an ORAC_OH_ assay (as µmol quercetin eq./g extract), and the capacity of the extract (200 µg/mL) to deplete nitric oxide (% depletion). All the results are presented as the mean ± SD.

	TBARS	ORAC_OH_	% NO^·^ Depletion
scSFE	45.13 ± 10.84	189.10 ± 0.81	29.37 ± 0.01

**Table 6:** Determination of the inhibition (%) ± SD of collagenase, tyrosinase, elastase, hyaluronidase, and glycosylation by scSFE. ** (*p* < 0.01) and **** (*p* < 0.0001) indicate statistically significant differences compared to the corresponding untreated negative control; ns: not statistically significant.

	Percentage of Inhibition				
Extract	Collagenase	Tyrosinase	Elastase	Hyaluronidase	Glycosylation
scSFE	−11.76 ± 2.71 ^ns^	40.47 ± 19.35 ^ns^	164.11 ± 27.33 ^**^	90.80 ± 5.93 ^****^	51.76 ± 7.06 ^****^

## Supplementary Materials

The following are available online at https://www.mdpi.com/2076-3921/9/5/418/s1, Table S1. Percentage of photoprotection for 100 and 200 µg/mL of scSFE extract and 800–1200 J/m^2^ of UVB, normalized to its respective irradiated condition. These values were calculated as indicated previously. Table S2. Percentage of oxidative stress inhibition of 100 and 200 µg/mL scSFE extract against oxidative stress induced by UVA and UVB. Doses of 3 and 6 J/cm^2^ UVA and 800 and 1200 J/m^2^ UVB were used. These values were calculated as indicated previously.
