# Supplementary material for: Sweet Cherry Byproducts Processed by Green Extraction Techniques as a Source of Bioactive Compounds with Antiaging Properties
Source: Antioxidants (Basel). 2020 May 13;9(5):418. doi: 10.3390/antiox9050418 (PMC7278782; doi:10.3390/antiox9050418)
Supplement: Supplementary file 1 [file antioxidants-09-00418-s001.pdf]

**Table S1.** Percentage of photoprotection for 100 and 200 μg/mL of scSFE extract and 800–1200 J/m$^2$ of UVB, normalized to its respective irradiated condition. These values were calculated as indicated previously.

| **scSFE extract** | **Photoprotection (%)** | |
|---|---|---|
| | **UVB Dose** | |
| Concentration (μg/mL) | **800 J/m$^2$** | **1200 J/m$^2$** |
| 100 | 14.61 | 3.51 |
| 200 | 36.53 | 13.99 |

Table S2. Percentage of oxidative stress inhibition of 100 and 200 μg/mL scSFE extract against oxidative stress induced by UVA and UVB. Doses of 3 and 6 J/cm$^2$ UVA and 800 and 1200 J/m$^2$ UVB were used. These values were calculated as indicated previously.

| **scSFE Extract (μg/mL)** | **Oxidative Stress Inhibition (%)** | | | |
|---|---|---|---|---|
| | **UVA Dose** | | **UVB Dose** | |
| | **3 J/cm$^2$** | **6 J/cm$^2$** | **800 J/m$^2$** | **1200 J/m$^2$** |
| **100** | 43.38 | 44.12 | 18.87 | 36.51 |
| **200** | 82.42 | 78.69 | 48.34 | 66.33 |
